# *Mycobacterium marseillense* Infection in Human Skin, China, 2018

**DOI:** 10.3201/eid2510.190695

**Published:** 2019-10

**Authors:** Bibo Xie, Yanqing Chen, Jian Wang, Wei Gao, Haiqing Jiang, Jiya Sun, Xindong Jin, Xudong Sang, Xiaobing Yu, Hongsheng Wang

**Affiliations:** Institute of Dermatology, Chinese Academy of Medical Sciences and Peking Union Medical College, Nanjing, China (B. Xie,Y. Chen, W. Gao, H. Jiang, H. Wang);; Zhejiang Institute of Dermatology, Deqing, China (B. Xie, J. Wang, X. Jin, X. Sang, X. Yu);; Jiangsu Key Laboratory of Molecular Biology for Skin Diseases and STIs, Nanjing (Y. Chen, W. Gao, H. Jiang, H. Wang);; Suzhou Institute of Systems Medicine, Chinese Academy of Medical Sciences, Suzhou, China (J. Sun);; Centre for Global Health, Nanjing Medical University, Nanjing (H. Wang)

**Keywords:** nontuberculous mycobacteria, skin diseases, skin infection, *Mycobacterium marseillense*, *Mycobacterium avium* complex, China, bacteria, mycobacteria, 16S rRNA sequencing, antimicrobial resistance, tuberculosis and other mycobacteria, paranasal sinus infection, sinusitis, virulence factors

## Abstract

We describe a case of facial skin infection and sinusitis caused by *Mycobacterium marseillense* in an immunocompetent woman in China in 2018. The infection was cleared with clarithromycin, moxifloxacin, and amikacin. Antimicrobial drug treatments could not be predicted by genetic analyses; further genetic characterization would be required to do so.

*Mycobacterium marseillense* is a member of the *M. avium* complex (*1*) that has caused infections with lymphatic or pulmonary involvement sporadically in humans (*2*–*4*). We report *M. marseillense* infection involving facial skin in an immunocompetent woman in eastern China.

In April 2018, a 59-year-old woman was referred to our institute (Institute of Dermatology, Chinese Academy of Medical Sciences and Peking Union Medical College, Nanjing, China) for a 4-year history of an erythematous plaque with ulceration located on the right cheek. The primary lesion was a small erythematic patch that gradually developed into an asymptomatic ulcerative plaque (i.e., the plaque had no heat, swelling, pain, or pruritus). She also reported occasional bloody, purulent nasal discharge over the course of 2 years. Two years before visiting our hospital, cutaneous tuberculosis was suspected, so she received treatment for tuberculosis (rifampin, isoniazid, ethambutol, pyrazinamide) for 10 months. No obvious improvement was observed with this treatment. Her medical history was otherwise unremarkable.

On physical examination, an infiltrated erythematous plaque with yellow scales and crusts on the right cheek was visible (Figure, panel A). Routine laboratory tests showed no remarkable findings. The results of autoantibody and HIV tests were negative, and immune subset cell counts were unremarkable. Histologic examination showed infiltration of a large number of lymphocytes, plasma cells, and neutrophils and some tissue cells in the dermis (Appendix 1 Figure 1). Computed tomography scan of the paranasal sinuses showed bilateral maxillary, right ethmoid, and frontal sinusitis (Figure, panel C). Culture and PCR for mycobacteria in nasal discharge yielded negative findings.

**Figure:**
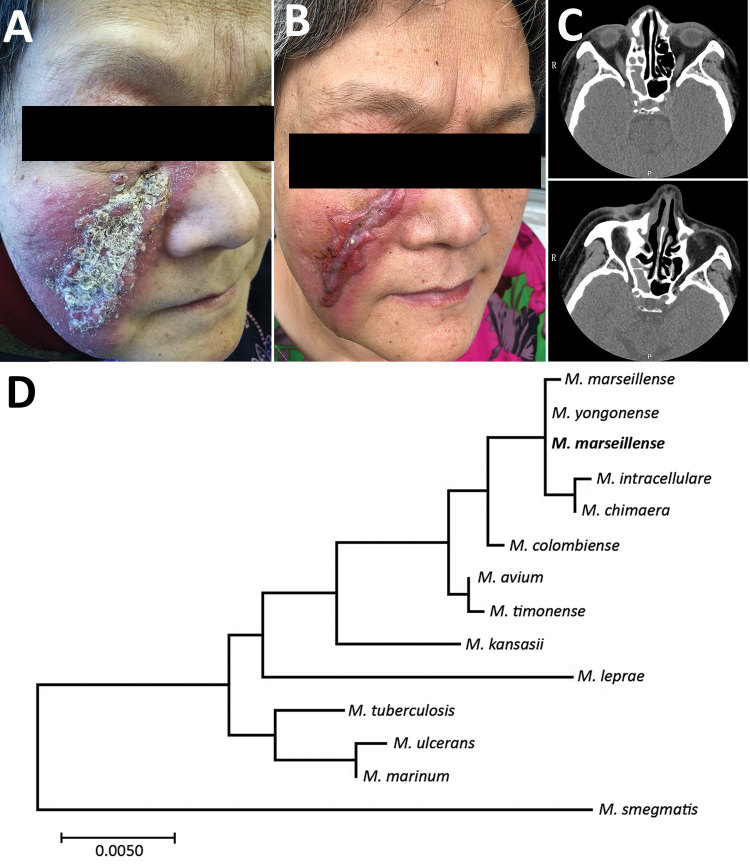
Skin lesions and computer tomography scans of woman with *Mycobacterium marseillense* skin infection, China, 2018, and genomic analysis of isolate. A, B) Facial skin lesion of woman with *M. marseillense* infection before and after treatment. Infiltrated erythematous plaque with yellowish scales and crusts (A) resolved to a scar after clearance of infection (B). C) Computed tomography imaging before treatment (top) shows heterogeneous hypersignal in right ethmoid sinus and after treatment (bottom) shows recovery of right ethmoid sinus. P, posterior; R, right. D) Phylogenetic tree constructed with 16S rRNA gene sequence of isolate from patient (bold) and other species. Scale bar indicates nucleotide substitutions per site.

After 3 weeks of skin tissue culture at 32°C in Löwenstein–Jensen medium, we observed smooth, yolk-yellow bacterial colonies (Appendix 1 Figure 2). Ziehl-Neelsen staining confirmed the cultured organism was acid-fast bacilli. Sequence analysis indicated that the complete genetic sequence of 16S rRNA was 99.0%, *hsp65* 100%, and *rpoB* 99.8% homologous with *M. marseillense* strain FLAC0026. Phylogenetic analysis of the 16S rRNA sequence showed the isolate clustered with *M. chimaera* and *M. intracellulare* (Figure, panel D). Although the 16S rRNA gene sequence of the isolate was 100% similar to *M. intracellulare* subsp. *yongonense* 05-1390, the sequence similarities to *hsp65* and *rpoB* were relatively low. Sequence analyses suggested *M. marseillense* infection.

Referring to the guidelines for pulmonary *M. avium* complex disease, we treated the patient with the antimicrobial drugs clarithromycin, rifampin, and ethambutol (*5*). Afterward, in vitro drug susceptibility testing showed the isolate was sensitive to clarithromycin, azithromycin, and amikacin; moderately sensitive to moxifloxacin; and resistant to ethambutol and rifampin. Therefore, 3 months after initiating treatment, we changed the regimen to clarithromycin, moxifloxacin, and amikacin, which she received for 2 months. The patient’s skin lesions healed gradually, and nasal symptoms disappeared, but a scar and erythema remained (Figure, panel B). Computed tomography scans of the paranasal sinuses showed the reduction of sinusitis (Figure, panel C). No recurrence was observed during 4 months of monitoring.

We characterized this isolate’s genome (GenBank accession no. VASI0000000) further to help determine the cause of its virulence and resistance (Appendix 1 Figure 3). Genetic analyses indicated the genome (≈5,706,022 bp) contained 5,343 predicted genes, 3 rRNAs, and 48 tRNAs and had a GC content of 67.73%. We annotated the genes functionally through multiple databases (Appendix 1 Table 1, Figure 4). Using the Virulence Factors of Pathogenic Bacteria database, we identified 137 potential virulence genes (identity >95.0%, E value <1 × 10^–5^), such as type VII secretion system genes (e.g., *esxH*, *esxC*, *esxH*, and *esxC*) (*6*), in the isolate’s genome (Appendix 2). In Comprehensive Antibiotic Resistance Database searches, we detected the antimicrobial drug resistance genes *mtrA*, *murA*, and *gyrA* (identity >90.0%, E value <1 × 10^–5^; Appendix 1 Table 2); *mtrA* modulates antimicrobial drug efflux, *murA* encodes the fosfomycin resistance protein, and *gyrA* encodes the fluoroquinolone resistance protein.

*M. marseillense* infections are rare in humans. Our case demonstrates that *M. marseillense* can cause infections in immunocompetent persons. For facial skin infection with *M. marseillense*, this and similar (*7*) reports indicate the need for vigilance of paranasal sinus infection. Although many potential virulence factors could be detected by genomic analysis, cases of infection and transmission with this bacterium are rarely reported, suggesting the presence of other influencing factors.

The drug resistance mechanisms of *M. marseillense* have not been completely elucidated. The drug susceptibility test results and treatment response we observed were generally consistent with those previously reported for cases of pulmonary infection, although sensitivity to rifampin and quinolones yielded various results (*2*–*4*). Drug susceptibility testing indicated that the isolate we obtained was resistant to ethambutol and rifampin. However, in genetic analyses, mutations associated with ethambutol and rifampin resistance were not detected. According to the Comprehensive Antibiotic Resistance Database, our isolate was resistant to fluoroquinolone, but drug susceptibility test results were inconsistent. Our results indicate that drug susceptibility testing should be performed for *M. marseillense* to guide antimicrobial drug treatment. If drug susceptibility results are absent, treatments including macrolides and amikacin appear to be reasonable.

Appendix 1 Additional information on *Mycobacterium marseillense* infection in human skin, China, 2018.

Appendix 2 Potential virulence genes (n = 137) identified in isolate found in skin lesion of patient, China, 2018, by using the Virulence Factors of Pathogenic Bacteria database.
