# Supplementary material for: Mycobacterium marseillense Infection in Human Skin, China, 2018
Source: Emerg Infect Dis. 2019 Oct;25(10):1991–3. doi: 10.3201/eid2510.190695 (PMC6759254; doi:10.3201/eid2510.190695)
Supplement: Appendix 1 — Additional information on Mycobacterium marseillense infection in human skin, China, 2018. [file 19-0695-Techapp-s1.pdf]

Article DOI: https://doi.org/10.3201/eid2510.190695

Mycobacterium marseillense Infection in Human Skin, China, 2018

Appendix 1

Material and Methods

Ethic Statement

Informed consent was obtained from the patient. Ethics approval was obtained from the ethics review board of the Institute of Dermatology, Chinese Academy of Medical Sciences, Nanjing, China.

Tissue Culture, Bacterial Strains, Genomic DNA Extraction and Sequencing

Skin tissue grinding fluid and nasal discharge from the patient were collected and cultured on Löwenstein–Jensen medium at 32°C and 37°C. The grown colonies of bacteria on Löwenstein–Jensen medium were then carefully transferred to Middlebrook 7H10 agar with Middlebrook oleic albumin dextrose catalase (OADC) enrichment and cultured at 37°C. *Mycobacterium marseillense* used in our study was isolated from the skin tissue of our patient. Genomic DNA Isolation was performed using a DNeasy Blood & Tissue kit (QIAGEN, The Netherlands) according to the manufacturer’s instructions. The DNA purity and concentration was measured by NanoDrop 2000 spectrophotometer. Genome sequencing was conducted by Novogene Technology Co. Ltd (Beijing, China) using Illumina PE150.

Genome Analyses

Circular genome maps were generated using Circos (Version 0.64) based on analysis of the coverage of sample sequencing reads, single-nucleotide polymorphisms (SNPs), and InDels. The coding genes were predicted by GeneMarkS software. CRISPRdigger was used to predict the CRISPRs (clustered regularly interspaced short palindromic repeats) of the genome. Scattered repeat sequences were predicted by RepeatMasker software, and TRF searched for tandem repeats in DNA sequences. Functional annotations were performed using the non-redundant protein (Nr) database, SwissProt, the Kyoto Encyclopedia of Genes and Genomes (KEGG) database, Cluster of Orthologous Groups of proteins (COG), Gene Ontology (GO), and protein fami-lies (Pfam). Antimicrobial resistance genes were detected based on the Antibiotic Resistance Genes Database (ARDB) and Comprehensive Antibiotic Resistance Database (CARD). Virulence factor annotation was carried out based on Virulence Factors of Pathogenic Bacteria database (VFDB) and Pathogen-Host Interactions database (PHI).

Phylogenetic Analyses

The phylogenetic tree was built based on the 16S rRNA gene sequence of *M. marseillense* in this study and other reference strains, including *Mycobacterium intracellulare* ATCC 13950, *Mycobacterium avium* subsp. paratuberculosis K-10, *Mycobacterium chimaera* strain AH16, *Mycobacterium marseillense* strain FLAC0026, *Mycobacterium colombiense* CECT 3035, *Mycobacterium intracellulare* subsp. *yongonense* 05–1390, *Mycobacterium timonense* CCUG 56329, *Mycobacterium kansasii* ATCC 12478, *Mycobacterium leprae* TN, *Mycobacterium tuberculosis* H37Rv, *Mycobacterium ulcerans* Agy99, *Mycobacterium marinum* E11, and *Mycobacterium smegmatis* strain MC2 155. The gene sequences were downloaded from the NCBI.

Appendix Table 1. Information on *Mycobacterium marseillense**

Sample ID	nr	SwissProt	KEGG	COG	TCDB	GO	PHI	VFDB	ARDB	CARD	Secretory protein	T3SS	CAZy
M. marseillense	5,220	2,309	5,164	3,836	295	3,467	292	454	0	27	108	186	140

*ARDB, Antibiotic Resistance Genes Database; CARD, Comprehensive Antibiotic Resistance Database; CAZy, Carbohydrate-Active enZYmes Database; COG, Cluster of Orthologous Group; GO, Gene Ontology; ID, identification; KEGG, Kyoto Encyclopedia of Genes and Genomes; NR, redundant protein database; PHI, Pathogen-Host Interactions database; T3SS, type 3 secretion system; TCDB, Transporter Classification Database; VFDB, Virulence Factors of Pathogenic Bacteria database.

Appendix Table 2. Antimicrobial resistance genes found in *Mycobacterium marseillense* isolate from patient, China, 2018*

ORF ID	Best Hit e value	Best Hit ARO	Best Identities	ARO	ARO name	Bit score	AR0 category
M. marsei GM004178, locus = Scaffold 16: 52255:52917:-	2.35E-157	mtrA	1	ARO: 3000816	mtrA	436.032	efflux pump conferring antibiotic resistance, gene modulating antibiotic efflux
M. marsei GM003653, locus = Scaffold 12: 152238:153491:+	0	Mycobacterium tuberculosis murA	0.96875	ARO: 3003784	Mycobacterium tuberculosis murA	813.142	fosfomycin resistance protein
M. marsei GM000974, locus = Scaffold 3: 140161:142677:+	0	Mycobacterium tuberculosis gyrA conferring resistance to fluoroquinolones	0.932934	ARO: 3003295, ARO: 3003941	Mycobacterium tuberculosis gyrA conferring resistance to fluoroquinolones, Shigella flexneri parC conferring resistance to fluoroquinolones	1572.76, 386.726	antibiotic resistant gene variant or mutant, fluoroquinolone resistance protein

*ARO, antibiotic resistance ontology; ID, identification; ORF, open reading frame.

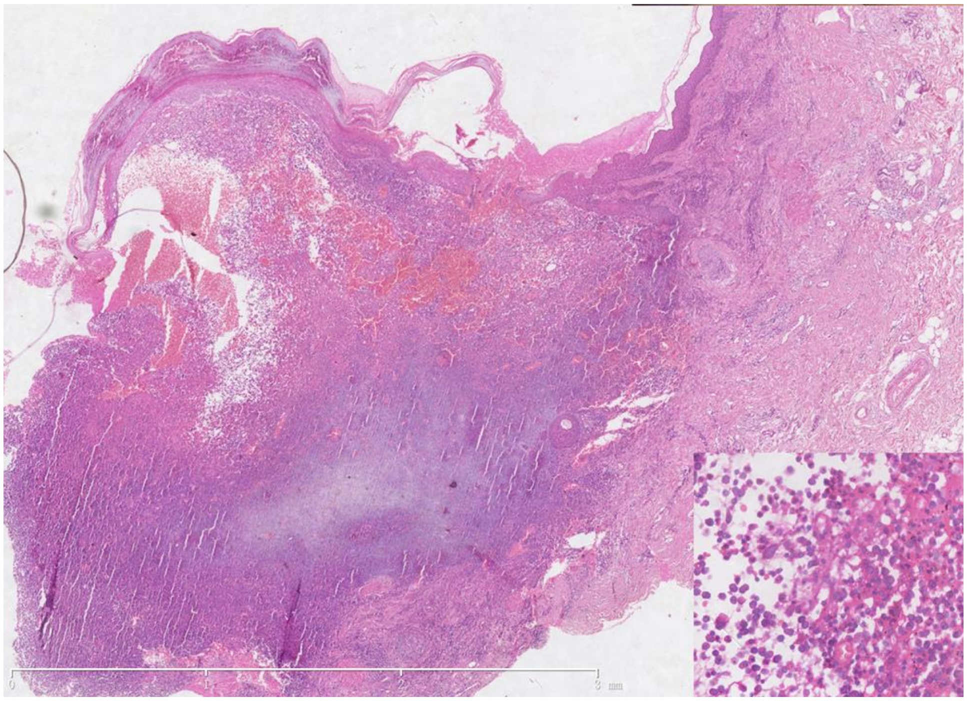

Appendix Figure 1. Hematoxylin and eosin stain of skin lesion (original magnification ×4). Inset shows part of the sample.

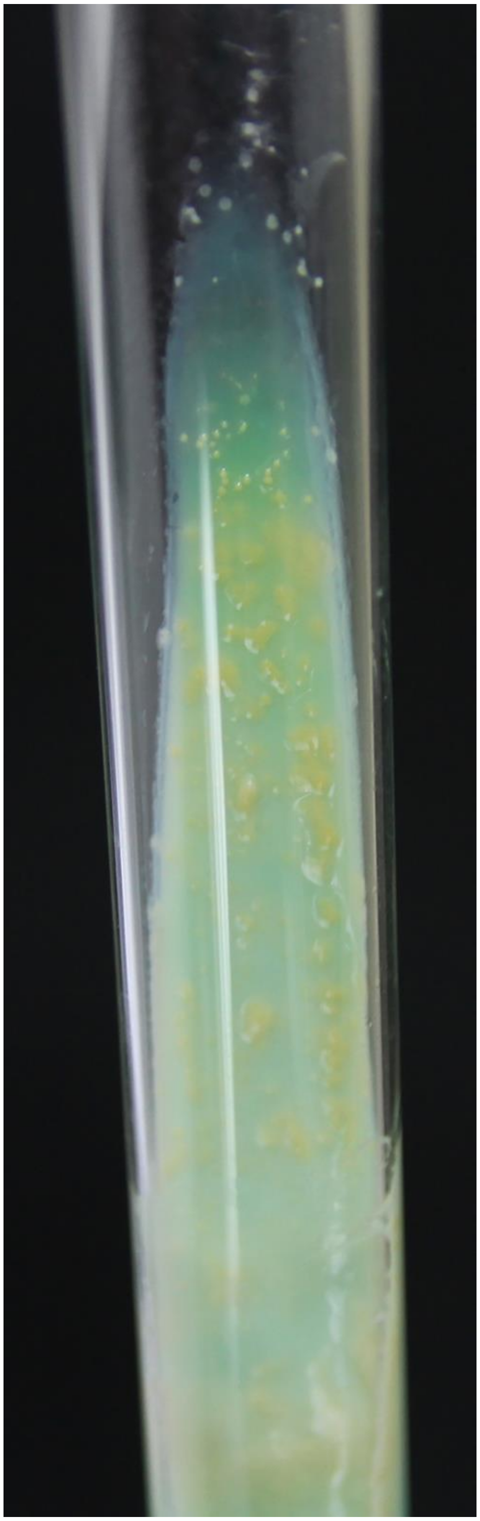

Appendix Figure 2. Tissue homogenate culture showing bacterial colonies after incubation on Löwenstein–Jensen medium.

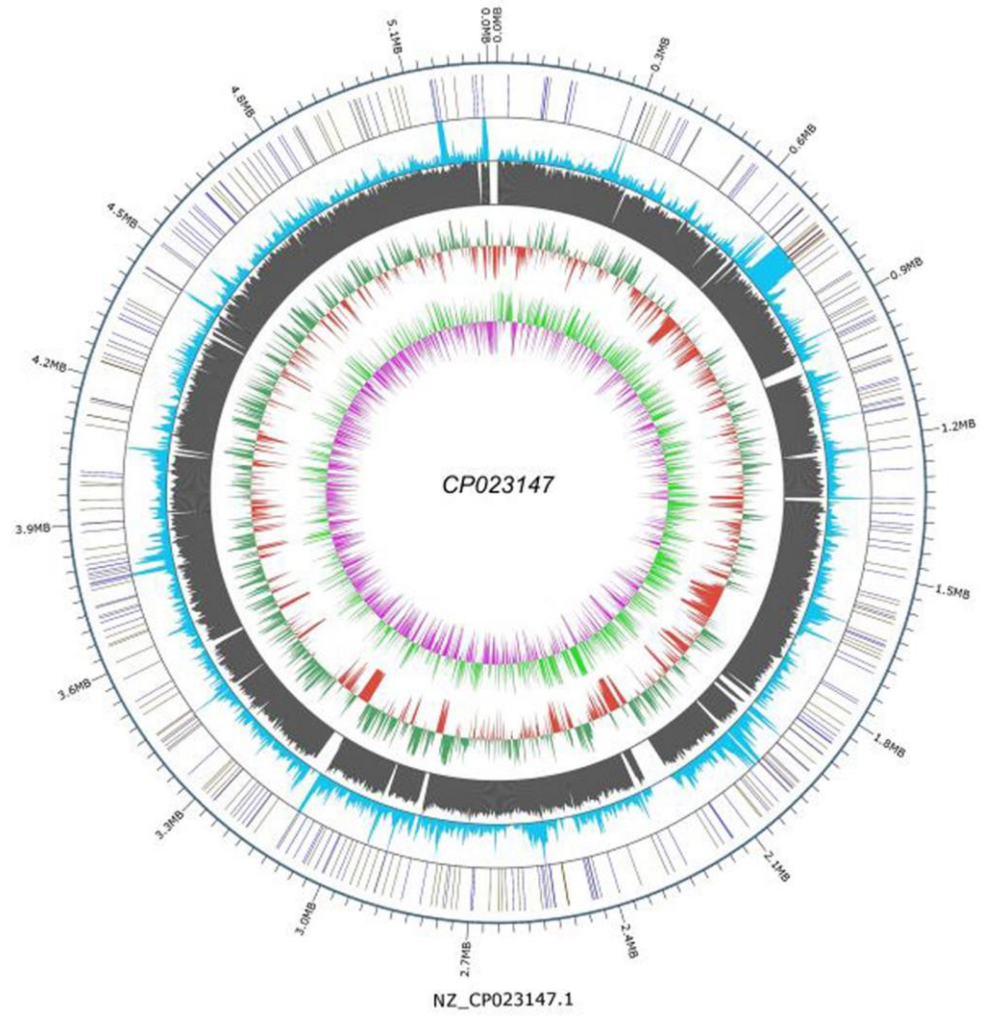

Appendix Figure 3. Overview of *Mycobacterium marseillense* genome. From the outermost to the innermost circle: the position coordinate of the reference sequence, the InDel distribution of the sample, the single-nucleotide polymorphism (SNP) number distribution of the sample, the reads coverage depth of the sample, the GC content of the reference sequence genome, and the GC skew value distribution of the reference sequence genome, respectively.

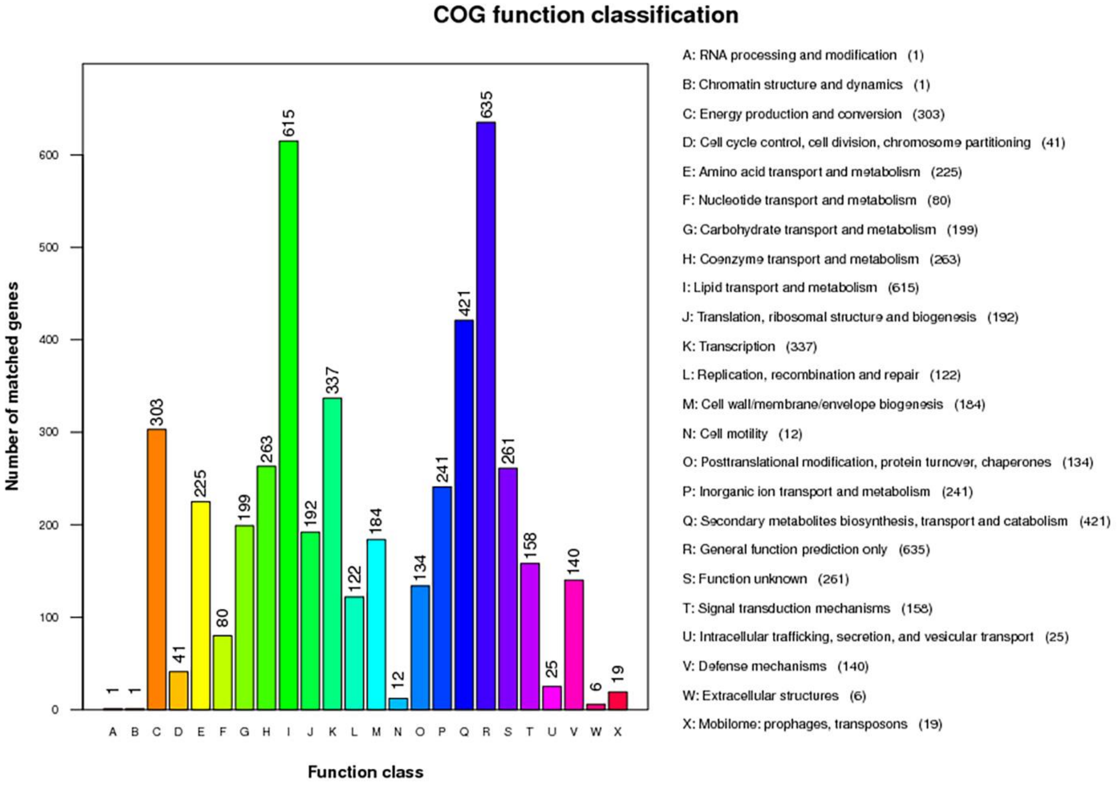

Appendix Figure 4. Function categories of genes by Cluster of Orthologous Group annotation.
